# Current Evidence on Endoscopic Biliary Drainage in the Era of Surgically Altered Anatomy: A Narrative Review

**DOI:** 10.3390/medicina62091766

**Published:** 2026-09-14

**Authors:** Davide Scalvini, Carlo Ciccioli, Angelo Bruni, Marco Valvano, Gianmaria La Rosa, Michele Dota, Alessandro Cappellini, Giulio Massetti, Guglielmo Aprile, Francesca Torello Viera, Letizia Veronese, Gianluca Franchellucci, Stefano Mazza, Aurelio Mauro, Marco Bardone, Alessandro Fugazza, Marco Spadaccini, Alessandro Repici, Andrea Anderloni

**Affiliations:** 1 Department of Internal Medicine and Therapeutics, University of Pavia, 27100 Pavia, Italy; 2 Gastroenterology and Digestive Endoscopy Unit, Fondazione IRCCS Policlinico San Matteo, 27100 Pavia, Italy; 3 Gastroenterology and Digestive Endoscopy Unit, ASST Rhodense, Rho Hospital, 20017 Rho, Italy; 4 Department of Medical and Surgical Sciences, University of Bologna, 40138 Bologna, Italy; 5 Gastroenterology Unit, IRCCS Policlinico di Sant’Orsola, Azienda Ospedaliero-Universitaria di Bologna, 40138 Bologna, Italy; 6 Gastroenterology Unit, E.O. Ospedali Galliera, 16128 Genoa, Italy; 7 Gastroenterology Unit, ASL Novara, 28100 Novara, Italy; 8 Digestive Endoscopy Unit, IRCCS Humanitas Research Hospital, 20089 Rozzano, Italy; 9 Department of Biomedical Sciences, Humanitas University, 20072 Milan, Italy

**Keywords:** surgically altered anatomy, biliary drainage, ERCP, endoscopic ultrasound, EUS-guided biliary drainage, enteroscope-assisted ERCP, hepaticogastrostomy, EDEE, EDGE, Roux-en-Y gastric bypass

## Abstract

Endoscopic retrograde cholangiopancreatography (ERCP) remains the reference standard for biliary drainage, yet it fails in up to 15% of cases, most notably when a surgically altered anatomy (SAA) is present. The expanding use of bariatric and oncologic gastrointestinal surgery has increased the number of patients in whom the papilla is displaced, unreachable, or replaced by a biliodigestive anastomosis, making conventional access difficult or impossible. This narrative review appraises the current evidence on endoscopic biliary drainage in SAA, focusing on procedural efficacy, anatomical peculiarities, adverse-event profiles and technical considerations to guide clinical decision-making. Available strategies include luminal techniques, duodenoscope-assisted ERCP, forward-viewing (cap-assisted colonoscope) ERCP, and enteroscope-assisted ERCP, as well as endoscopic ultrasound-guided biliary drainage (EUS-BD), encompassing EUS-guided hepaticogastrostomy, EUS-guided antegrade stenting, EUS-guided rendezvous and EUS-directed transgastric/transenteric ERCP (EDGE/EDEE), and laparoscopic-assisted ERCP (LA-ERCP). No single approach is universally superior and available comparative data is mainly retrospective and affected by major selection bias. Technique selection should be tailored to the reconstruction type, indication (benign vs. malignant), limb length, expected survival, and local expertise, ideally within a multidisciplinary, high-volume setting. Adequately powered randomized trials incorporating quality-of-life and cost-effectiveness endpoints are still needed to define the optimal first-line strategy. This literature review encompasses the multiple strategies to achieve biliary drainage in surgically altered anatomy, highlighting strengths and weaknesses of each technique, as well as the preferred approach for each type of anatomical reconstruction.

## 1. Introduction

Endoscopic retrograde cholangiopancreatography (ERCP) is still considered the gold standard for biliary drainage, especially in the setting of benign disease [1,2]. Nevertheless, a large Dutch cohort, including both biliary and pancreatic indications, reported ERCP failure in up to 15% of the cases, caused by a variety of technical and anatomical factors, among which the presence of a surgically altered anatomy (SAA) represents one of the most significant [3,4].

Surgically altered anatomy represents a considerable challenge for endoscopists; in many of them, the papilla is not “native” anymore or displaced in a different position in the duodenum. Moreover, surgical reconstruction may result in a substantially increased distance between oral cavity and the papilla or in the creation of a biliodigestive anastomosis that precludes conventional luminal endoscopic access [5,6,7,8,9,10].

For all these reasons, ERCP frequently fails in the context of SAA and several other techniques have been proposed to overcome these limitations [11,12,13,14,15,16].

The selection of the most appropriate approach depends mainly on the clinical indication and the specific type of surgical reconstruction. The variety of available endoscopic techniques could be summed in luminal techniques, Endoscopic ultrasound (EUS)-guided or EUS-assisted techniques, and laparoscopic-assisted technique. Luminal techniques encompass those strategies in which biliary access is achieved via the papilla or biliodigestive anastomosis using a duodenoscope, a standard, long or cap-assisted colonoscope, or a single- or double-balloon enteroscope (enteroscope-assisted ERCP, EA-ERCP) [17,18,19].

Other techniques include laparoscopic-assisted ERCP (LA-ERCP), which is a well-established and highly effective strategy in difficult biliary drainage in SAA; however, it is associated with significant technical complexity, as well as logistical and resource constraints [20].

EUS-guided or EUS-assisted biliary drainage (EUS-BD) has emerged as a promising alternative, although its adoption remains largely confined to tertiary referral and highly specialized centers [11,12,13,14,15,16].

A recent Italian survey highlighted that the availability of these techniques varies considerably across institutions, and that procedural choice is frequently driven by local resource availability and individual operator experience rather than by standardized protocol [12]. Additionally, percutaneous transhepatic biliary drainage (PTBD) continues to be widely adopted, despite its well-recognized weaknesses, including the risk of infectious complications and the adverse impact on patients’ quality of life [21,22,23].

In recent years, the use of EUS-BD techniques has substantially increased, driven by the growing prevalence of obesity and the widespread adoption of surgical techniques (e.g., Roux-en-Y gastric bypass), as well as the verified efficacy on normal anatomy and by the improved long-term survival of patients undergoing Whipple’s pancreaticoduodenectomy for distal malignant biliary obstruction [24,25,26,27,28].

The expansion of EUS-BD has been further facilitated by its established efficacy in patients with normal anatomy, which has enabled the development of innovative approaches applicable to SAA. These include transmural antegrade stenting or EUS-guided hepaticogastrostomy (EUS-HGS), performed even when the endoscope cannot reach the papilla or bilioenteric anastomosis directly [29,30,31]. In the EUS-assisted technique, EUS-transmural approach creates a fistulous communication between the gastric remnant and the enteric limb (EUS-directed transgastric ERCP, EDGE) or between two enteric limbs (EUS-directed transenteric ERCP, EDEE), thereby circumventing the anatomical distance imposed by surgical reconstruction [11,12,13,14,15,16].

The most recent guidelines by the European and American endoscopy societies recommend that the advanced techniques used in SAA be reserved for high-volume expert centers following multidisciplinary decision-making. While these guidelines endorse EDGE and EA-ERCP as the preferred first-line approaches, their recommendations are based on low-quality evidence and were published several years ago, with a substantial body of literature having appeared since. An updated review is therefore warranted, both to integrate this new evidence and to define the uncertainties that persist [32,33].

In a clinical landscape in which no single technique has demonstrated clear superiority over the others, a rigorous appraisal of the advantages and limitations of each approach is essential to inform clinical decision-making, standardize patient management strategies and identify unmet needs warranting further investigation.

Accordingly, this review provides an expert, narrative evaluation of the existing literature on endoscopic biliary drainage in the context of SAA, with particular focus on procedural efficacy, adverse event profiles, technical considerations and practical algorithm.

## 2. Anatomical Implications of Surgically Altered Anatomy

To guide the endoscopic approach to biliary drainage, SAA may be classified into two distinct categories based on the anatomical preservation of duodenal continuity [5,6,34,35].

### 2.1. Type I SAA

Type I includes reconstructions in which the duodenum remains in continuity with the gastric remnant, preserving a relatively physiological route to the native ampulla of Vater. In this setting, luminal techniques or standard duodenoscope-assisted access is generally feasible, and the main issue is usually scope stability rather than access itself.

• Sleeve gastrectomy, performed for the treatment of obesity, consists of resection of the greater curvature of the stomach with preservation of continuity between the gastric remnant and the duodenum.
• Billroth I gastrectomy (BI), typically performed for gastric cancer or peptic ulcer disease, consists of antrectomy followed by an end-to-end gastroduodenostomy.

In both cases, the papilla remains in its native position and ERCP can generally be performed using a side-viewing duodenoscope and conventional devices, although there may be some loss of scope stability due to a shorter position and gastric geometry.

### 2.2. Type II SAA

Type II includes all reconstructions in which the stomach is absent or no longer in continuity with the duodenum [34]. In these patients, biliary access is limited by long intestinal limbs, sharp anastomotic angulations, altered endoscopic orientation, or replacement of the native papilla with a surgically created biliodigestive anastomosis. Type II anatomy can be further divided into two subgroups depending on the presence of a native ampulla of Vater.

#### 2.2.1. Altered Anatomy with Native Ampulla of Vater

**Figure 1:**
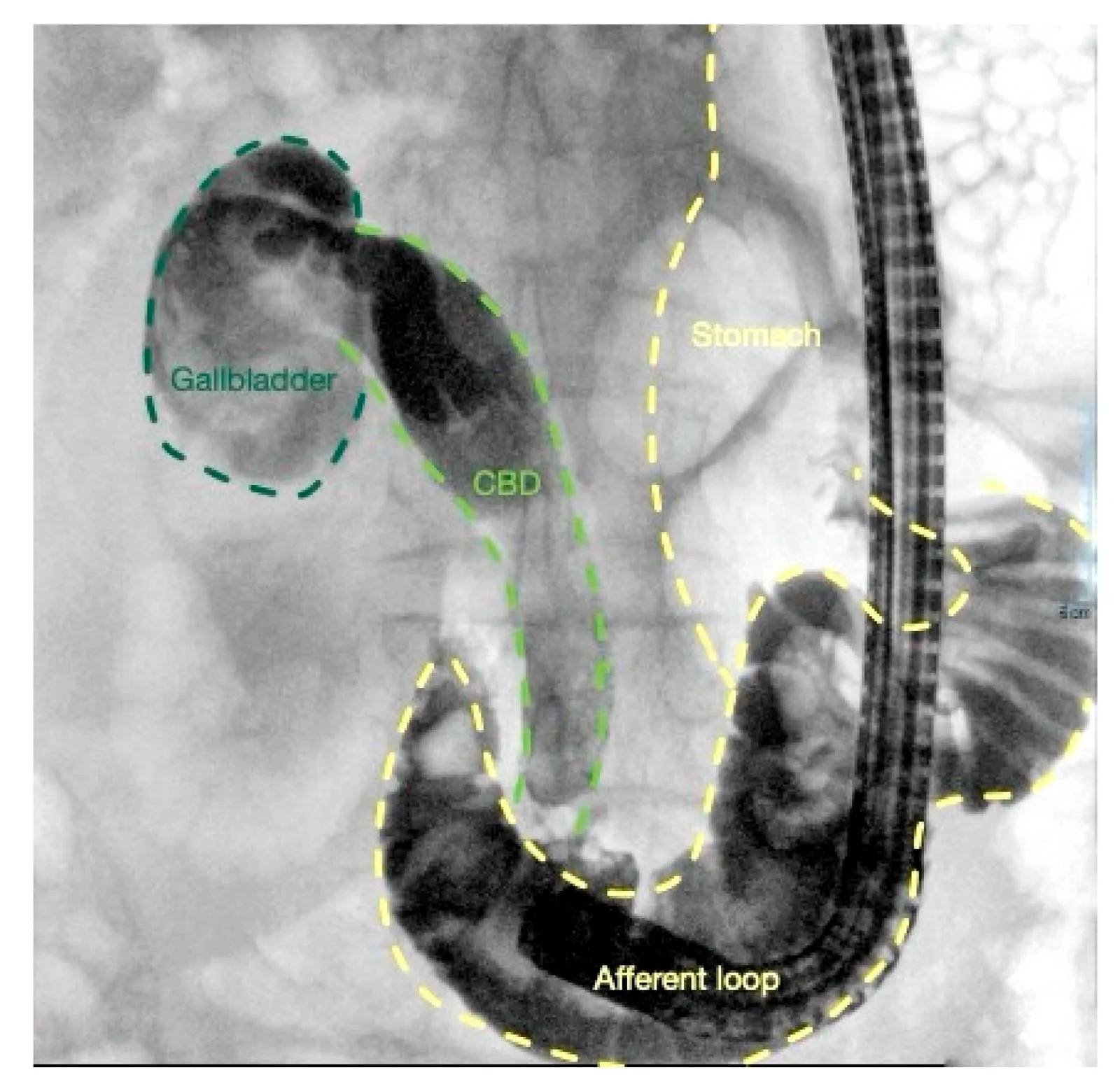
Fluoroscopic image of biliary drainage in Billroth II anatomy.

**Figure 2:**
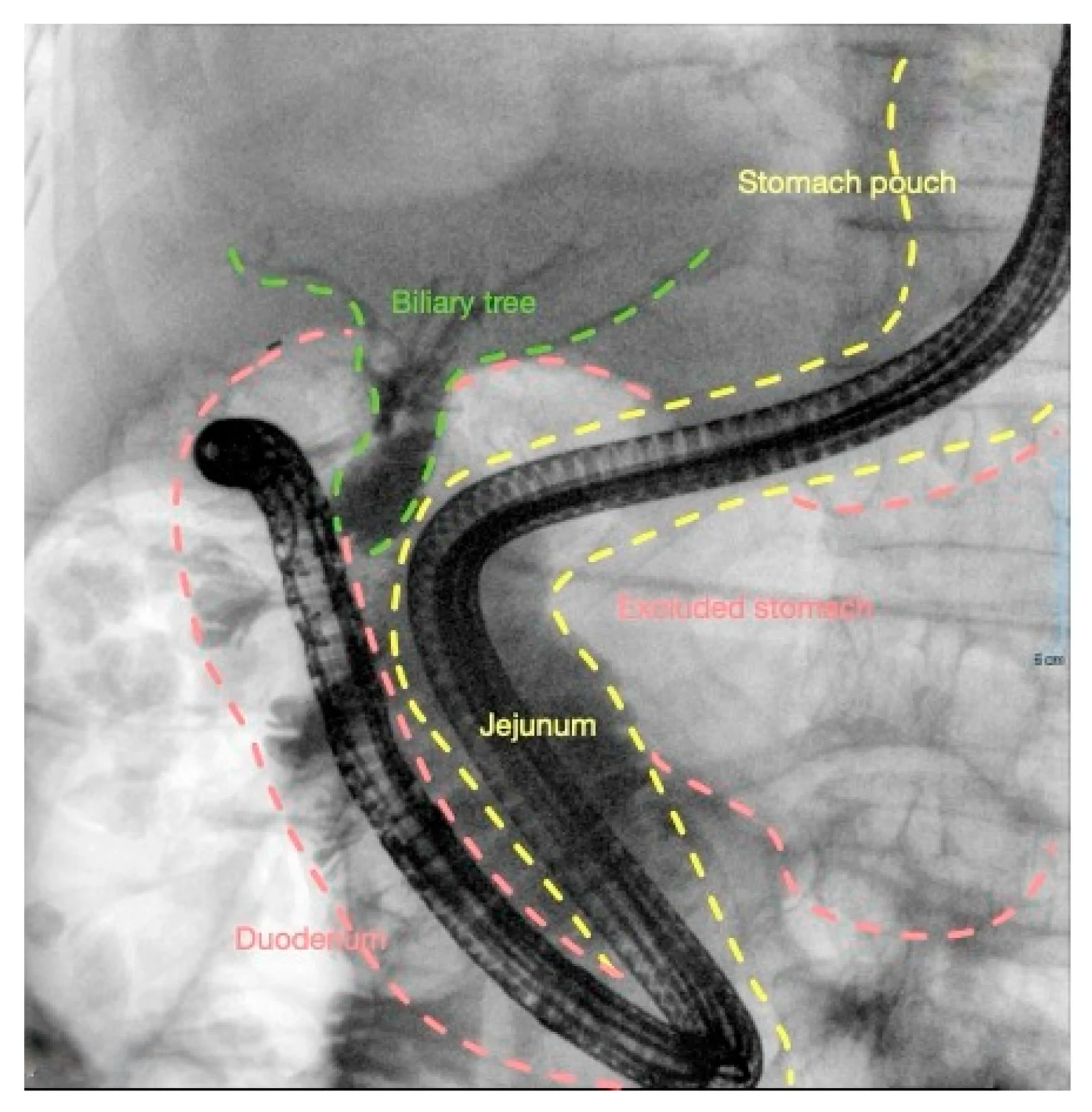
Fluoroscopic image of biliary drainage after Roux-en-Y reconstruction.

• Billroth II gastrectomy (B-II) and gastrojejunostomy were performed in the past for gastric cancer or peptic ulcer disease. The reconstruction is usually a side-to-end gastrojejunostomy, which creates an afferent limb in continuity with the duodenum and an efferent limb for alimentary transit. The afferent limb is typically 30–50 cm long. The papilla is approached through the afferent loop in an inverted orientation, with the biliary axis usually at the 5–6 o’clock position rather than the conventional 11–12 o’clock position. The Braun anastomosis is a modification of the B-II reconstruction that introduces an additional entero-enterostomy to connect the afferent and efferent intestinal loops. B-II anatomy is particularly challenging and may carry a higher risk of perforation, especially when a side-viewing duodenoscope is used [11]. Moreover, it may necessitate specialized devices, such as reverse sphincterotome [7,17,36]. Figure 1 shows a biliary cannulation during B-II.
• Roux-en-Y gastric bypass (RYGB) is a bariatric procedure for obesity in which a small proximal gastric pouch is created and the excluded stomach remains in continuity with the duodenum. The duodenum and proximal jejunum form the biliopancreatic limb, while the alimentary stream passes through the Roux limb, which is connected to the pouch through a gastrojejunostomy. The Roux limb is usually 75–150 cm long, and the biliopancreatic limb is joined to it through a jejunojejunostomy with a lower length ranging approximately from 30 to 75 cm [26,27]. Papillary access is difficult because the native papilla remains in the excluded duodenum and must be reached luminally through a long, angulated, multi-anastomotic route [27]. Figure 2 shows a biliary cannulation with forward colonoscope during short-limb RYGB.
• Mini gastric bypass/one-anastomosis gastric bypass (OAGB) is a single-loop variant that does not require the creation of a Roux limb or a jejunojejunostomy. A long gastric pouch is anastomosed directly to the small bowel through a single gastrojejunostomy, resulting in an afferent biliopancreatic limb and an efferent alimentary limb [4]. The papilla remains excluded from the alimentary stream, so access remains anatomically complex.
• Subtotal or total gastrectomy with Roux-en-Y reconstruction is used for gastric malignancy or severe benign disease. In subtotal gastrectomy, continuity is restored by a gastrojejunostomy to a Roux limb; in total gastrectomy, by an esophagojejunostomy. The jejunojejunostomy is usually located 35–60 cm distal to the primary anastomosis. The main technical issue is long-limb traversal, together with sharp angulation and loss of direct orientation toward the papilla [4].

#### 2.2.2. Altered Anatomy with Biliodigestive Anastomosis

This subgroup includes patients in whom bile drainage no longer occurs through the native papilla, but through a surgically created biliodigestive anastomosis [6].

**Figure 3:**
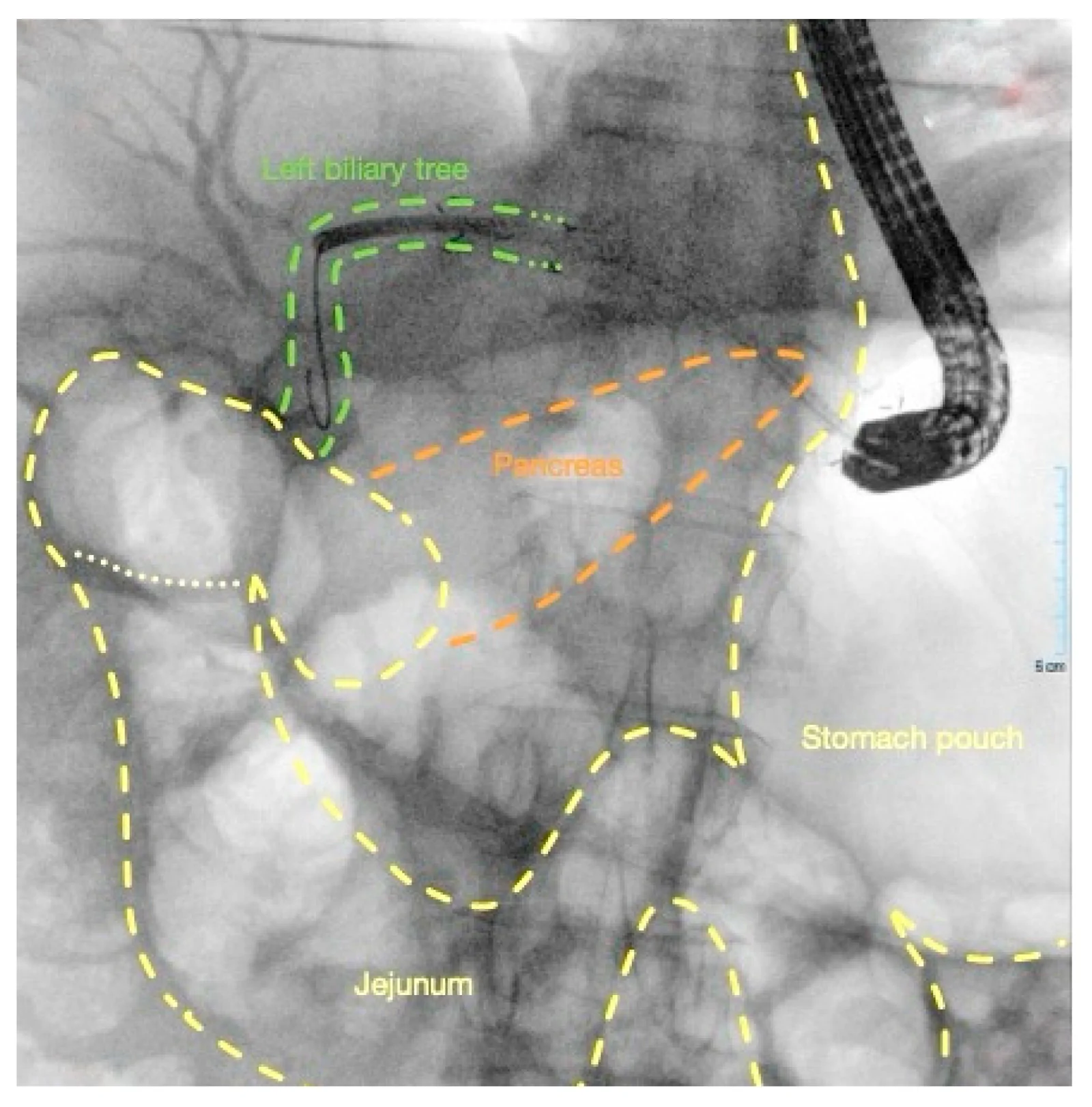
Fluoroscopic image of biliary drainage after pancreaticoduodenectomy.

• Pancreaticoduodenectomy (Whipple’s procedure) is performed mainly for periampullary and pancreatic head malignancies. Reconstruction requires a pancreaticojejunostomy, a hepaticojejunostomy and a gastrojejunostomy or duodenojejunostomy in pylorus-preserving variants. The hepaticojejunostomy is usually located 5–10 cm downstream from the pancreaticojejunostomy. In addition, alongside the classic technique involving antral resection, a pylorus-preserving approach has been described as facilitating jejunal intubation. Technical difficulty arises from multiple anastomoses, postoperative adhesions, loop fixation, and the need to identify a biliary anastomosis that is often approached obliquely and may be difficult to stabilize [4,8,11]. Figure 3 shows a EUS-guided hepaticogastrostomy after failed papilla cannulation in Whipple’s reconstruction.
• Roux-en-Y hepaticojejunostomy is performed for bile duct injury, choledochal cyst excision, liver transplantation, or other hepatopancreatobiliary reconstructions. The stomach and duodenum are usually intact, but the proximal bile duct is anastomosed to a jejunal limb through a hepaticojejunostomy. The jejunojejunostomy is usually 15–20 cm distal to the ligament of Treitz, and the biliary anastomosis is often near the end of a blind afferent limb or cul-de-sac, making identification and cannulation technically demanding.

Across Type II reconstructions, biliary access is limited by limb length, anastomotic angulation, inverted orientation, and reduced scope stability. As a result, conventional ERCP remains feasible in most Billroth II cases, while in the remaining type II SAA it is considerably more demanding, its outcome being influenced by the reconstruction performed, the length of the afferent limb and the target to be reached.

## 3. Luminal Techniques for Biliary Drainage in SAA

### 3.1. Duodenoscope-Assisted ERCP

Conventional duodenoscope-assisted ERCP can be used in Billroth’s reconstructions and after sleeve gastrectomy. In these reconstructions, especially with a relatively short afferent limb (<50–60 cm), a standard side-viewing duodenoscope can be the right choice, allowing for the use of an elevator, a larger working channel and conventional ERCP accessories. The largest single-center Billroth II experience, with more than 1000 ERCPs, reported a successful cannulation in 93.8% of the patients [17]. Adverse events occurred in 4.3%, with afferent-limb perforation in 1.8%, confirming that the access phase, rather than the therapeutic phase, is the major vulnerability of this approach. A more recent pooled analysis, evidenced a 95.3% rate of cannulation with side-viewing duodenoscope with a rate of adverse events of 7.9% and bowel perforation of 3.6%, increased compared the rate for forward viewing endoscope (1.7%) [37]. First-time ERCP and Braun anastomosis were independent predictors of technical failure [38].

Few strategies may be used to increase safety and efficacy. Allocate the patient in the left lateral decubitus can help to entry into the afferent loop. Contrast injection helps define the angle of the limb; the scope should then be advanced cautiously under fluoroscopy rather than forced. Use different devices such as reverse sphincterotome or straight cannula for cannulation and inflated balloon or overtube stabilizing and shortening loops. More recently, rigidizing overtubes have emerged as supplementary tools to support progression toward the papilla in altered-anatomy ERCP. They work by minimizing loop formation and enhancing scope stability when traversing long or tortuous afferent limbs. One such device, the Pathfinder overtube, is an 85 cm single-use instrument that can be introduced in a flexible configuration and subsequently rigidized via vacuum activation, achieving up to a 15-fold increase in stiffness [39]. Nevertheless, the available evidence on these novel devices remains limited and their role as standard instruments has yet to be established, so they should still be considered emerging tools.

### 3.2. Forward-Viewing ERCP

**Figure 4:**
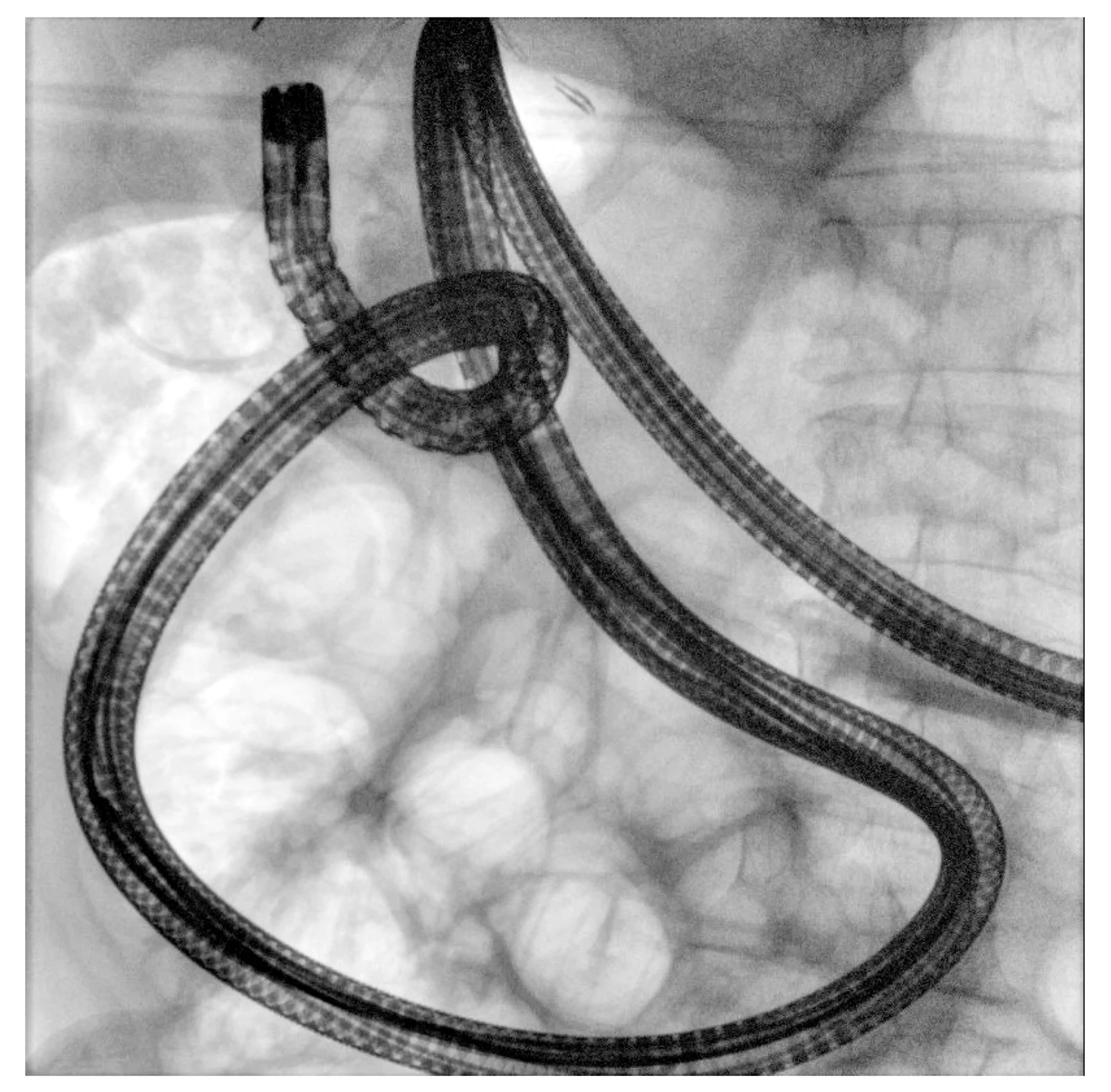
The use of “long” colonoscope in Roux-en-Y reconstruction.

Long afferent limbs, sharp angulations, adhesions and reversed papillary orientation may impair access, cannulation, and therapeutic completion turning in a failure of the conventional side-viewing duodenoscope approach. In this setting, forward-viewing endoscopes represent a pragmatic luminal alternative when duodenoscope-assisted ERCP fails or in Roux-en-Y reconstruction [11,40]. Pediatric colonoscope or “long” colonoscope may be used for this purpose: their forward orientation facilitates afferent-limb identification and progression through tortuous or angulated segments, while their shorter working length compared with long enteroscopes preserves broader compatibility with standard accessories and provides a working channel compatible with almost all devices (Figure 4). The principal limitation is the absence of an elevator, which makes cannulation of an inverted or tangentially oriented papilla more challenging. This drawback can be mitigated by careful retroflexion when feasible and, more consistently, by cap-assisted cannulation, which improves tip stability, maintains an adequate working distance, flattens mucosal folds, and facilitates alignment with the biliary axis [41,42]. In B-II anatomy, systematic review data showed high performance for forward-viewing endoscopy in afferent-limb intubation of 97.4% without impairment of the selective cannulation (95.2%), together with a lower bowel perforation rate than duodenoscope attempt (1.7% vs. 3.6%) [37]. However, this systematic review showed a higher rate of post-ERCP pancreatitis with forward-viewing endoscope compared to side-viewing duodenoscope (5.4% vs. 2.5%) [37].

Additional data support routine cap use, as cap-fitted forward-viewing endoscopes achieved higher afferent-limb intubation and cannulation success [41,42]. Moreover, cap-fitted pediatric colonoscope-assisted ERCP was evaluated in 93 patients with Roux-en-Y gastrectomy and native papilla, showing high endoscope insertion success of (88.17%) and cannulation success (85.37%) with an adverse event rate of 7.53% and no perforation or bleeding [43].

Lastly, underwater insertion has also been proposed to reduce loop formation through buoyancy and minimize overdistension [44].

These findings support forward-viewing colonoscope-based ERCP as a valid first-line in B-II or Roux-en-Y anatomy with short-limb when EUS-techniques and EA-ERCP are unavailable. However, in long-limb Roux-en-Y anatomy, reachability remains the main limiting factor and timely escalation to device-assisted enteroscopy or EUS-guided drainage is often required [11].

### 3.3. Enteroscope-Assisted ERCP

Enteroscope-assisted ERCP (EA-ERCP), including ballon-enteroscopy ERCP (BE-ERCP), either double-balloon (DBE) or single-balloon (SBE) and more selectively spiral-assisted platforms, is one of the main luminal approaches for long-limb SAA, particularly Roux-en-Y gastric bypass, hepaticojejunostomy and pancreaticoduodenectomy [11,18,19,31,45]. Short-type enteroscopes, with a working length of approximately 152–155 cm and a 3.2 mm channel, have improved compatibility with standard ERCP accessories compared with conventional 200 cm enteroscopes, making stone extraction, stenting and dilation feasible once the papilla or bilioenteric anastomosis are reached [46,47].

**Figure 5:**
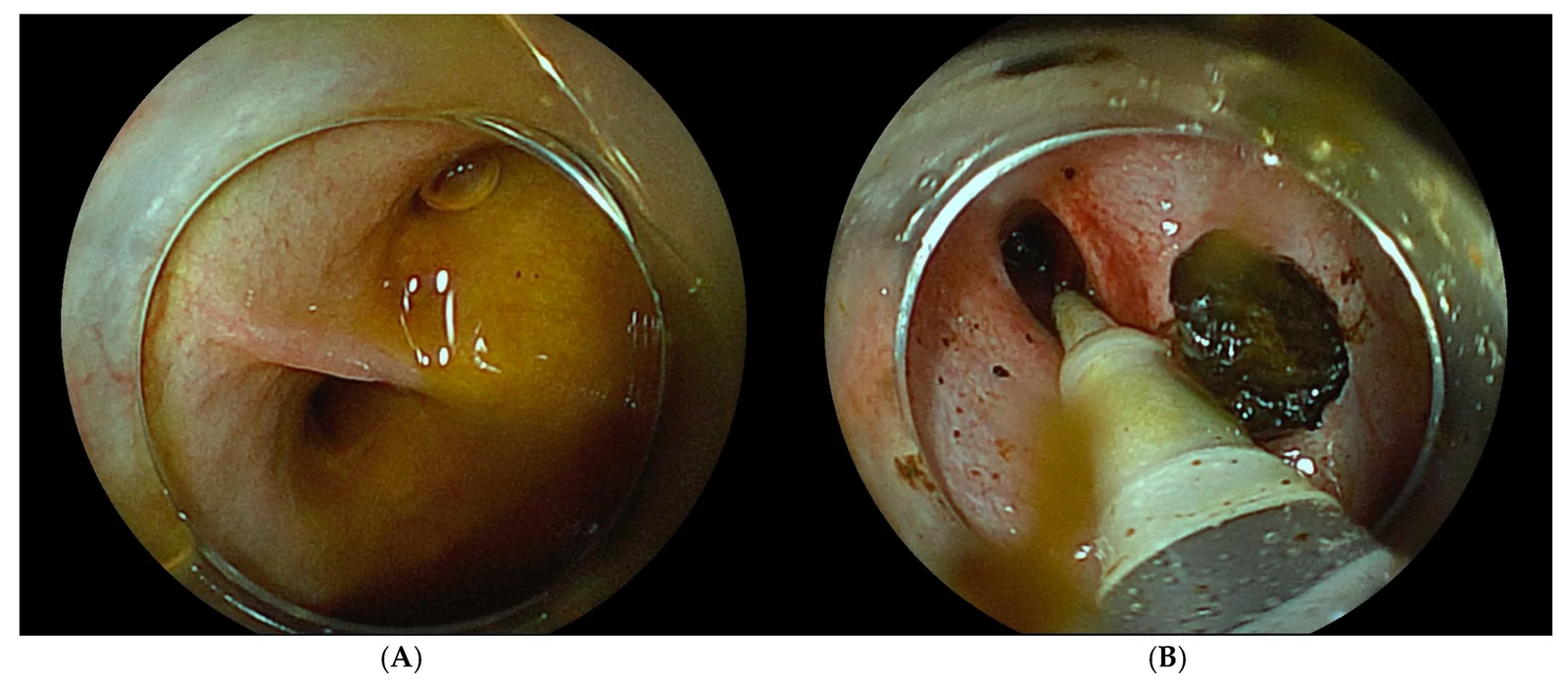
((**A**), on the left) EA-ERCP evaluating a double duct Roux-en-Y hepaticojejunostomy; ((**B**), on the right) EA-ERCP stone retrieval from a double duct Roux-en-Y hepaticojejunostomy.

Technically, BE-ERCP relies on sequential balloon inflation and deflation to pleat the small bowel, with CO_2_ insufflation and fluoroscopic guidance to identify the afferent limb. In native papilla cases, cannulation is challenging because the biliary axis is often shifted toward the 5–6 o’clock position; cap-assisted forward cannulation, papillary “hooking,” and retroflex or J-turn positioning can improve alignment (Figure 5A,B).

Available data highlighted the efficacy of BE-ERCP in SAA, the highest for B-II and decreased for Roux-en-Y [11,15]. Among different enteroscopes, large retrospective data suggest comparable success rates for intubation, cannulation, procedure completion and adverse events between SBE and DBE [46]. However, SBE required significantly longer intubation (23.5 vs. 14.1 min) and total procedure times (65.2 vs. 31.0 min). Moreover, SBE showed higher rates of post-ERCP cholangitis and cholecystitis.

Rigidizing overtubes, Endorail^®^ and spiral platforms may improve stability and reach, but current evidence remains limited and these adjuncts should be considered case-specific rather than standard of care [45,48,49].

BE-ERCP remains time-consuming and highly anatomy-dependent. Roux-en-Y reconstruction, benign indication, pancreatic indications, younger age, very long limbs and complex prior surgery have been associated with lower success. Adverse events are related not only to access difficulty but also to multiple cannulation attempts, papillary balloon dilation, and bile duct or papillary biopsies. Table 1 shows the main features of luminal techniques for biliary drainage in SAA.

**Table 1:** Main features of luminal techniques for biliary drainage in SAA.

	Working Length	Operative Channel	Accessory Compatibility
**Duodenoscope-assisted ERCP**	~124 cm	4.2 mm	Full; elevator available
**Forward-viewing (colonoscope) ERCP**	133–168 cm (pediatric/standard), up to 200 cm (“long”)	3.2 mm (pediatric); 3.7 mm (standard/”long”)	Broad for long or standard; no elevator, cap-assisted cannulation compensates
**Enteroscope-assisted ERCP**	152–155 cm (short-type); 200 cm (conventional)	3.2 mm (short-type); 2.8 mm (conventional)	Limited: short-type improves compatibility; conventional 200 cm scopes require dedicated extra-length devices

## 4. EUS-Guided or EUS-Assisted Biliary Drainage in SAA

EUS-guided biliary drainage has emerged as a valid first-line or rescue therapy in case of failed ERCP for distal malignant biliary obstruction [24,25,29,30,31]. This increased use has allowed for the introduction of EUS-guided biliary drainage approach in SAA as an efficient alternative to forward-viewing endoscopes or DAE-ERCP [11,29,30,50,51].

The most recent published European Society of Gastrointestinal Endoscopy (ESGE) guideline have tried to standardize the terminology about therapeutic EUS procedures defining two groups: “EUS-assisted” procedures (indirect technique) and “EUS-guided” interventions (direct technique) [33]. In the first category (“EUS-assisted” procedures), ESGE refers to the use of EUS to enable another procedure: techniques allowing for the possible creation of communication between the stomach and the gastric remnant in the Roux-en-Y gastric bypass (endoscopic ultrasound-directed transgastric ERCP or EDGE), or between the biliary loop in Roux-en-Y hepaticojejunostomy and entero-enteric anastomosis (endoscopic ultrasound-directed transenteric ERCP or EDEE) [50,51,52,53,54]. This anastomosis is created between the placement of a lumen-apposing metal stent (LAMS) and the subsequent execution of the ERCP through the LAMS. By contrast, “EUS-guided” interventions involve only the use of a linear echoendoscope and are performed exclusively under EUS guidance.

EUS-guided biliary drainage techniques include EUS-guided rendezvous (RV), EUS-guided antegrade [EUS-AG] stent placement or EUS-guided hepaticogastrostomy (EUS-HGS). All EUS-BD procedures are associated with four major steps: puncture of the bile duct (usually with a 19G needle), guidewire passage, transmural tract dilation (using a cystotome or a balloon) and stent placement. All these procedures require high skills and knowledge considering their high difficulty and rates of AEs of up to 12.8% [55,56,57].

To date, the only systematic review and meta-analysis present in the literature on EUS-guided biliary drainage in SAA managed to aggregate 18 studies (15 retrospective, three prospective; 12 monocentric, six multicentric with cohorts between 12 and 110 patients) for a total of 409 patients. The pooled technical success, clinical success, and adverse events rate were 97.8%, 94.9% and 12.8% (95% CI, 7.4–18.1%), respectively [55]. Moreover, the definitions of technical success, clinical success and adverse events likely varied across the included studies and should be acknowledged as a limitation. However, the correct use of EUS-BD in SAA should be tailored based on the patient’s anatomy, the underlying problem, expected survival and local expertise.

### 4.1. EUS-Guided Hepaticogastrostomy

First performed by Marc Giovannini in 2001, this procedure starts with the identification of a dilated left intrahepatic bile duct branch (segment 2 or 3 of left liver) from proximal stomach (or jejunum depending on surgical reconstruction), puncturing with a 19G needle and injecting with contrast to confirm the position, and a guidewire—either straight or angulated—is advanced in the intrahepatic duct. The tract is then dilated using a 6 Fr cystotome or balloon and, subsequently, a stent (usually a dedicated partially covered metallic stent or a fully covered metallic stent) is placed to create the hepaticogastrostomy [29,58,59,60,61].

EUS-HGS performs well in surgically altered anatomy precisely because it bypasses the problem that limits luminal techniques: it does not require the navigation in the reconstructed tract, which makes it a suitable second-line approach.

**Figure 6:**
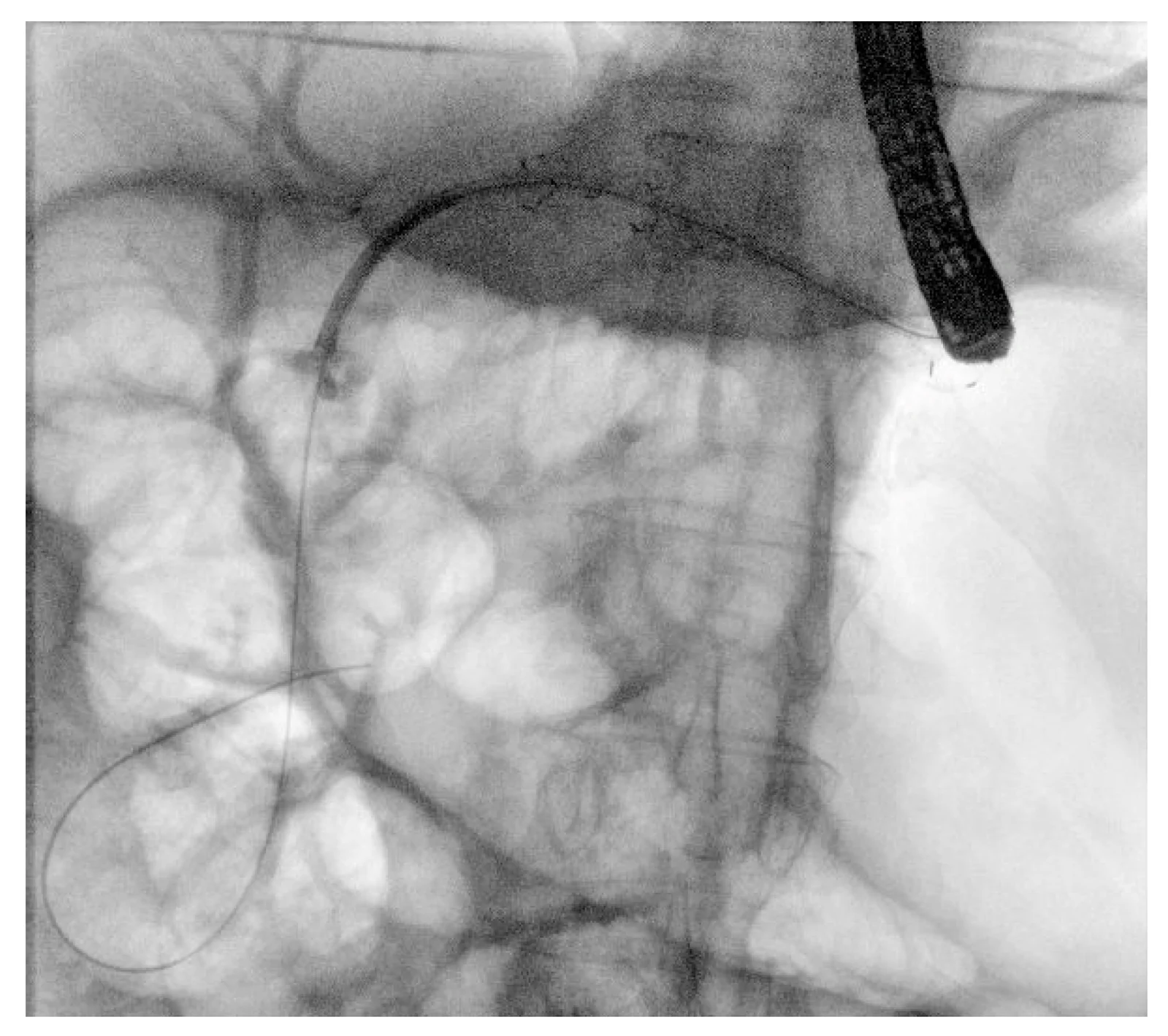
Cholangioscopic-assisted guidewire passage through a biliodigestive stricture in Roux-en-Y hepaticojejunostomy after EUS-guided hepaticogastrostomy with partially covered SEMS placement.

In a recent meta-regression, the pooled technical and clinical success of EUS-HGS were 97.7% and 88.1%, but just few reports are available for SAA specifically [62]. A single-center retrospective Japanese study compared EUS-guided hepaticogastrostomy (*n* = 29) with single-balloon enteroscopy-assisted ERCP (*n* = 41) in 70 patients with malignant biliary obstruction and SAA. EUS-HGS showed a higher technical success rate (100% vs. 68%), shorter procedure time (23 vs. 50 min), and longer stent patency (251 vs. 103 days), with higher but non-statistically differente AEs (20.6% vs. 17.1%) [31]. Moreover, another Japanese multicenter study showed higher technical success rate of EUS-BD, with the majority of the procedure performed via EUS-HGS, compared to EA-ERCP (94% vs. 70%), with comparable clinical success and AEs rate characterized mainly by cholangitis, bleeding, bile leak and abdominal pain [13]. However, the choice of EUS-HGS is burdened by its non-negligible rate of AEs and the often irreversible nature of the procedure related to long-term stent indwelling or the creation of a permanent fistula [56]. Given that the conditions associated with SAA are frequently benign in nature, such as stenosis of biliodigestive anastomosis, the use of EUS HGS as a first-line approach remains debatable and its risk–benefit ratio should be carefully weighed (Figure 6). On the contrary, when available, EUS-HGS may be considered a first line solution in malignant diseases when papilla/anastomosis is not reachable. Nevertheless, some authors propose that the definitive route may enable follow-up treatment where necessary, as in difficult biliary stones, with no larger infection risk than PTBD [14].

### 4.2. Antegrade Stenting

**Figure 7:**
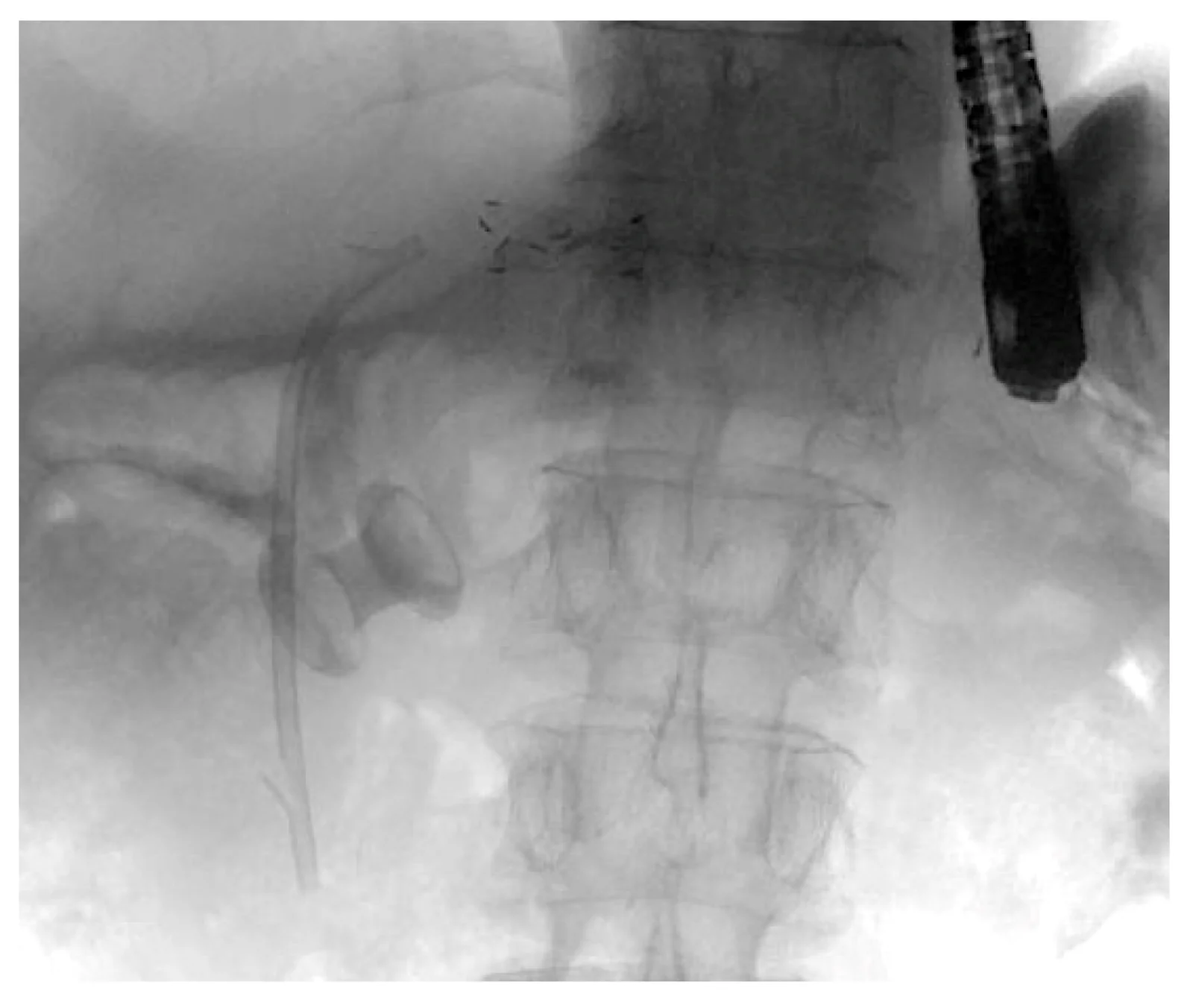
Combined EUS-guided hepaticogastrostomy with partially covered SEMS and transpapillary EUS-guided antegrade stenting with plastic stent after failed EUS-directed trans-enteric ERCP for biliodigestive stricture in Roux-en-Y hepaticojejunostomy.

First described by Nguyen-Tang in 2010, this technique involves the puncturing of left intrahepatic bile duct branch either from the stomach or the duodenum, with subsequent progress of a guidewire to the papilla or bilioenteric anastomosis. The tract could be dilated, a biliary stone managed with an extraction balloon or a transpapillary stent placed across a stricture in an antegrade fashion [63]. The main drawback is the need to create a fistulous tract between the stomach and the liver. In a large series of EUS-AS placement including patients with SAA, technical success reported was 88.7%, with clinical success of 95.7% [64]. Lastly, pairing EUS-HGS with transpapillary EUS-AS may offer an advantage over a single drainage method, although this remains an open debate with insufficient data yet (Figure 7) [65,66,67].

### 4.3. EUS-Guided Rendezvous ERCP

First described by Mallery in 2004, this technique is very useful in cases where the papilla or the bilioenteric anastomosis are accessible with the scope, but deep cannulation is not possible, in particular in benign diseases [68]. An intrahepatic duct, or rarely the common bile duct, is punctured from the stomach or enteric limb under endoscopic ultrasound guidance and a guidewire is advanced through the dilated biliary system along the papilla or anastomosis. The instrument is then withdrawn, leaving the guidewire in place. At this point, a duodenoscope is switched in to conclude the procedure, catching the guidewire or with side-by-side technique. In rare cases, access through the gallbladder has been reported, whereby a previously placed LAMS from EUS-guided gallbladder drainage is used to reach the common bile duct via the cystic duct and perform a rendezvous procedure [69].

A recent meta-analysis supports the effectiveness of this technique following unsuccessful biliary cannulation, with ERCP reporting a pooled technical success rate of 86.1%, a clinical success rate of 80.8%, and an overall adverse event rate of 14%, including cholangitis, bile leakage, and bleeding [70]. EUS-RV is safe and effective in SAA. Moreover, it should be preferred to PTBD considering the lower rate of post-procedural pain, sepsis, median hospital stay and stent dysfunction [71,72].

EUS-RV approach is most feasible in patients with Billroth II reconstruction or hepaticojejunostomy, where the papilla or the bilioenteric anastomosis remain at least potentially accessible.

### 4.4. EUS-Directed Trans-Gastric or Trans-Enteric ERCP (EDGE, EDEE)

EDGE and EDEE are particularly useful in surgical reconstructions with long limb such as RYGB and pancreaticoduodenectomy and in cases where repetitive interventions could be necessary, such as in benign biliary strictures or biliodigestive anastomosis stricture or difficult biliary stones [33,51]. In 2014 Kedia et al. described in patients with RYGB the first EDGE [53]. In a large systematic review, EDGE showed high technical success (96%) with a pooled adverse events rate of 17%, especially failure of fistula closure, stent migration and bleeding with a rate of perforation of 4% [73].

By contrast, EDEE has been introduced more recently and should therefore still be considered an emerging technique. It involves the creation of a gastro-enteric or entero-enteric shortcut, allowing endoscopic access to the biliary loop by using LAMS, therefore enabling ERCP. This is especially useful in patients in which EUS-HGS or EUS-AG was not feasible due to the small caliber of biliary ducts [52]. In a recent multicenter retrospective study, EDEE demonstrated high technical and clinical success rates of 87.3% and 93.8%, respectively. The overall adverse event rate was 20%, with 9.1% specifically related to LAMS [16].

**Figure 8:**
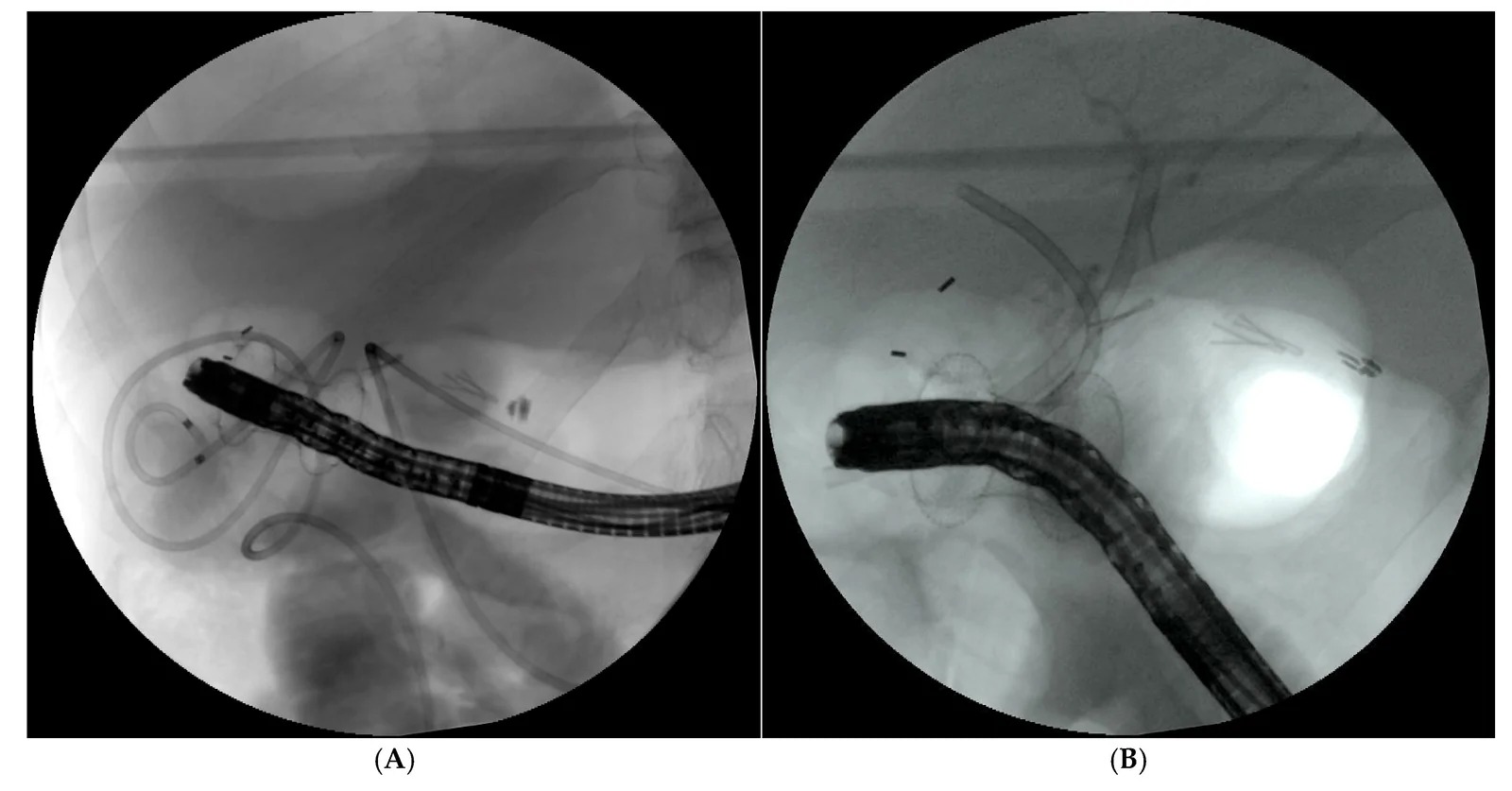
EDEE procedure for biliodigestive stricture in Roux-en-Y hepaticojejunostomy. ((**A**), on the left) Long-colonoscope move through an entero-enteric anastomosis just created with LAMS after naso-jejunal tube insufflation. ((**B**), on the right) Positioning of two straight plastic stent to dilate the biliodigestive stricture.

Few studies have investigated whether performed one step or dual session EDGE/EDEE after LAMS placement with controversial conclusions [74,75,76]; however, new data suggest that the anchoring of the LAMS and the use of larger LAMS, independent of the single or dual session, may reduce the risk of LAMS migration (Figure 8) [75,77,78]. In addition, the interval between LAMS placement and the subsequent EDGE/EDEE procedure should be tailored to the clinical urgency of the intervention and to the stent-fixation technique employed.

Systematic reviews and meta-analyses showed an increased technical success of EDGE compared to EA-ERCP and a lower length of stay and procedural time compared to EA-ERCP and LA-ERCP [79,80].

## 5. Laparascopic-Assisted ERCP

Laparoscopic-assisted ERCP (LA-ERCP) showed interest for several reasons: the possibility to perform a single session with concomitant cholecystectomy in the same operative session of the biliary drainage, taking advantage of the existing surgical access and secondly, the use of a duodenoscope through the trocar instead using a forward-view endoscope, and the high success rate. In addition, the technique may also be employed in an emergency setting for the management of postoperative biliary complications.

With the LA-ERCP, under general anesthesia the surgeon proceeds with the insertion of one or more trocars into the gastric remnant and after the placement of a gastric trocar fixed through the gastrostomy, the endoscopist introduces a side-viewing duodenoscope through the gastrotomy, allowing endoscopic progression along the physiological route to the papilla of Vater [81].

Large studies consistently demonstrate very high technical efficacy of LA-ERCP in RYGB patients. In a multicenter cohort of 579 patients, the overall procedural success rate was 98.1%, with similar rates reported in systematic analyses (95–96%) [82,83]. From a clinical outcome perspective, LA-ERCP is relatively safe but not without complications. Across multiple series and metanalysis, the overall 30-day adverse event rate ranges from 18% to 28%, especially mild to moderate AEs. The most common complications arise from both the endoscopic and surgical components, such as post-ERCP pancreatitis 3–7%, surgical site infections 3–9% and perforations (gastrotomy or small bowel) around 2–4% [81,82,83,84,85].

However, the principal drawback is its invasiveness: it is a combined surgical-endoscopic procedure that increases surgical morbidity and requires general anesthesia, an operating room and coordination between a surgeon and an endoscopist, with the associated logistical complexity and cost. Procedure times are long and scheduling is more cumbersome than a purely endoscopic alternative. Lastly, repeated biliary access after LA-ERCP may require a further surgical procedure unless a gastrostomy tract is maintained.

## 6. Techniques Comparisons and Discussion

Achieving effective biliary drainage in patients with SAA represents an increasing clinical challenge, driven by the rising prevalence of gastrointestinal surgical procedures mostly performed for both bariatric indications and oncological management [27,86]. As this review illustrates, no single technique is universally superior: the optimal approach depends on the type of surgical reconstruction, the length of the biliary limb, the underlying disease, endoscope availability, and local expertise [4,11,12]. Moreover, the vast majority of data was derived from retrospective studies, involving substantial confounding/bias by anatomy, indication, operator expertise and institutional selection.

Taken together, and in light of the considerations outlined above, EA-ERCP and EUS-assisted/-guided biliary drainage emerge as the two most effective and versatile techniques. These distinctions are reflected in the current guidelines, with ASGE suggesting EDGE over EA- or LA-ERCP in RYGB and EA-ERCP first in non-RYGB anatomy (reserving EUS-BD or PTBD for failure), while ESGE recommends EUS-BD as a first-line option only for malignant obstruction with a long, dilated biliary limb [32,33]. When they are compared directly, the retrospective literature consistently favors EUS-BD for technical performance. Khashab et al. reported higher technical and clinical success with EUS-BD than with EA-ERCP (technical success 98% vs. 65.3%; *p* = 0.001), with shorter procedures but more adverse events (AEs), while Hakuta et al. confirmed superior technical success in malignant obstruction, with comparable clinical success and AE rates [13,87]. The same trend emerges from real-world practice: in the large multicenter STREET study, overall technical and clinical success were broadly similar across reconstruction types. Moreover, the increasing use of interventional EUS over the final two years was independently associated with a markedly higher likelihood of clinical success and fewer reinterventions [11]. This signal has now been corroborated prospectively, as a multicenter registration trial of primary drainage for malignant SAA found EUS-BD to achieve significantly higher technical success and shorter procedure time than balloon-enteroscopy-assisted ERCP, particularly in Roux-en-Y anatomy, with comparable clinical success, AEs, and stent patency; balloon-enteroscopy-assisted ERCP was an independent predictor of technical failure, and the advantage of EUS-BD was highlighted in patients with Roux-en-Y reconstruction [13,88]. Included in the EUS-BD techniques EDGE is increasing its use. In a systematic review and meta-analysis, EDGE and laparoscopic-assisted ERCP achieved similarly high success, clearly exceeding EA-ERCP [89]. In more recent pooled data, technical success reached approximately 96% for EDGE and 93% for LA-ERCP versus approximately 77% for EA-ERCP (*p* < 0.05), whereas overall adverse-event rates were numerically higher for EDGE and LA-ERCP (20% and 19%) than for EA-ERCP (13%) without reaching statistical significance, underscoring an efficacy–safety trade-off that is central to technique selection in RYGB [85,90,91,92,93]. Taken together, the first prospective comparison now reinforces EUS-BD over EA-ERCP for malignant SAA, and real-world data show outcomes improving as practice shifts toward interventional EUS [11,12,88].

Beyond the choice of technique per se, both real-world and comparative data indicate that outcomes are strongly operator and volume-dependent: in the STREET study, the progressive use of EUS techniques across the study period independently predicted clinical success and fewer reinterventions [11], reinforcing the guidelines suggestion for concentrating these procedures in high-volume centers able to offer the full portfolio of all approaches for SAA. The higher efficacy of EDGE and LA-ERCP must therefore be weighed against their greater invasiveness and adverse-event burden, which supports their use in RYGB and when repeated biliary access is anticipated, whereas the lower adverse-event rate and full reversibility of EA-ERCP preserve its value as a less-invasive option in non-RYGB anatomy and in benign disease. Moreover, a cost-effectiveness model showed that EDGE was the most cost-effective modality in RYGB anatomy for the treatment of pancreaticobiliary diseases compared with LA-ERCP [90].

Purely luminal ERCP nonetheless retains a first-line role in simple, short reconstructions such as Billroth I–II and short afferent limbs, where, in experienced hands, duodenoscope- or forward-viewing-assisted access achieves high cannulation rates (>93%) [17,37]; given its minimal invasiveness and the fact that it leaves every subsequent option open, a luminal attempt remains a reasonable initial step whenever the papilla or bilioenteric anastomosis are endoscopically reachable, even at the price of a comparatively lower technical success. This apparent simplicity, however, must not be mistaken for a benign procedure. Real-world multicenter data are less reassuring than expert series: in the STREET study, the adverse-event rate was the highest precisely in Billroth II patients (14.4%) [11], an anatomy in which afferent-limb perforation is the characteristic serious complication [17,37]. Moreover, the “price” of a luminal-first strategy is not confined to procedural adverse events—it may also entail repeated sessions or longer procedures, with the cumulative sedation and anesthesia exposure and attendant risks that these imply. Taken together, the low threshold and apparent accessibility of a luminal attempt may foster a false presumption that these conditions can be treated in any setting; given the strong operator and volume-dependence of outcomes noted above, such cases are instead best centralized in high-volume, fully equipped referral centers. Table 2 summarize the main characteristics of each endoscopic drainage.

PTBD itself remains a reliable fallback, achieving high technical success, but the indwelling external catheter increases infection risk and reduces patient quality of life [71,72,92,93]. Meta-analyses show that EUS-BD matches PTBD technically while offering better clinical outcomes, fewer reinterventions and lower costs, whereas EA-ERCP requires fewer procedures and shorter treatment than PTBD for anastomotic strictures.

Evidence for benign disease and direct head-to-head data among EDGE, LA-ERCP, and PTBD nonetheless remain largely retrospective, and adequately powered randomized trials, stratified by reconstruction type and by benign versus malignant indication and incorporating quality-of-life and cost-effectiveness endpoints, are still needed to define the optimal first-line strategy.

**Figure 9:**
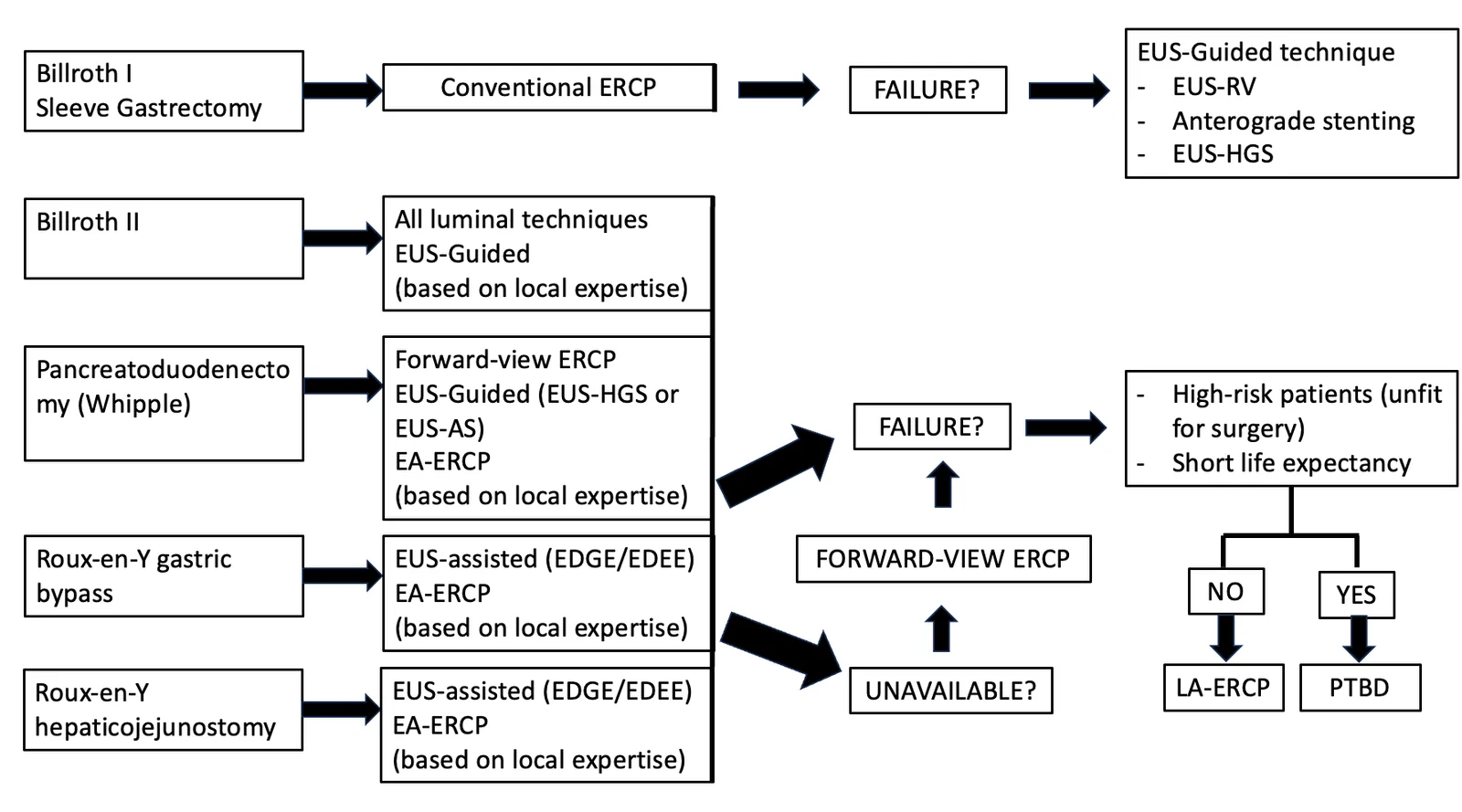
Proposed flowchart for biliary drainage in surgically altered anatomy.

On the basis of the available evidence, a tentative algorithm for biliary drainage in patients with SAA is proposed (Figure 9). In addition to the type of reconstruction, further variables should be considered, including hemodynamic stability, severity of cholangitis, degree of ductal dilation, presence of ascites, coagulopathy and the anticipated need for repeated biliary access.

## 7. Conclusions

Given the variety of surgical reconstructions, no single technique is universally effective and the choice should be anatomy- and indication-specific rather than an institutional habit. Success should be accordingly judged on more than achieving drainage in the fewest attempts. Safety, durability of drainage, quality of life and resource utilization, including operating-room requirements and cumulative sedation exposure, all belong in the assessment.

In Roux-en-Y gastric bypass, the evidence is the most consistent and favors EUS-BD compared to other techniques. EDGE is therefore a reasonable first-line option, particularly when repeated biliary access is anticipated, whereas LA-ERCP retains a role when concomitant cholecystectomy is planned, and EA-ERCP remains valuable where full reversibility is a priority.

Outside RYGB, the evidence base is weaker and the hierarchy differs. In Billroth I–II and short afferent limbs, luminal ERCP achieves high cannulation rates and, being minimally invasive and leaving every subsequent option open, remains a reasonable first step whenever the papilla or bilioenteric anastomosis is endoscopically reachable. However, the reported high rate of afferent limb perforation with conventional ERCP in B-II warrants mention.

The benign and malignant settings should be taken in consideration. In malignant obstruction, comparative data increasingly favor EUS-BD over EA-ERCP, with higher technical success and longer stent patency and without an excess of severe adverse events. In benign disease, where life expectancy is long and the irreversibility of an EUS-HGS tract carries lasting consequences, techniques that preserve future options may be preferable, and the supporting evidence remains considerably thinner.

Flexibility in using different biliary drainage technique is essential to personalize treatment of this group of patients. The most important aspect is the need to tailor the intervention in a case-by-case fashion, taking into account the surgery performed considering a multidisciplinary approach with tight collaboration among gastroenterologists, radiologists and surgeons. The choice of the procedure should also rely upon the expertise and equipment available at every institution given the long learning curve needed to perform such operations.

Finally, these recommendations derived from largely retrospective, often single-center, operator-dependent evidence, with heterogeneous outcomes and definitions of technical success, clinical success, and adverse events strongly influenced by procedural volume. Adequately powered randomized trials stratified by reconstruction type and by benign versus malignant indication, incorporating quality-of-life and cost-effectiveness endpoints, remain necessary to define the optimal first-line strategy.

## Figures and Tables

**Table 2:** Summarise of the main outcomes of each techniques applied for biliary drainage in surgically altered anatomy.

Technique	Main Anatomical Indication	Technical/Clinical Success	Adverse Events	Reversibility and Practical Notes
**Duodenoscope-assisted ERCP**	Type I SAA (sleeve, Billroth I); Billroth II with short afferent limb (<50–60 cm)	Billroth II: pooled cannulation 93.8–95.3%	AEs 4.3–7.9%, perforation 1.8–14.4% in B-II	Fully reversible; elevator and full accessory range available; access phase is the main vulnerability
**Forward-viewing (colonoscope) ERCP**	Billroth II; short-limb Roux-en-Y; salvage after failed duodenoscope	Billroth II: afferent-limb intubation 97.4%, selective cannulation 95.2%; RYGB insertion 88.2%, cannulation 85.4%	AEs 7.5%, Perforation 1.7%, PEP 5.4%	Fully reversible; no elevator, mitigated by cap-assisted cannulation; limited by reach in long limbs
**Enteroscope-assisted ERCP**	Long-limb SAA: RYGB, hepaticojejunostomy, pancreaticoduodenectomy	RYGB: pooled technical success ~77%; highest in Billroth II and lower in Roux-en-Y reconstructions.	Pooled AEs 13% in RYGB; SBE longer time and more post-ERCP cholangitis/cholecystitis	Fully reversible; least invasive of the advanced options; time-consuming and highly anatomy-dependent
**EUS-BD (overall in SAA)**	Failed or unfeasible luminal access or malignant	Mixed SAA and techniques: pooled technical 97.8%, clinical 94.9%	Pooled AEs 12.8%: cholangitis, bleeding, bile leak and abdominal pain	Requires advanced skills; evidence mostly retrospective and in non-SAA
**EDGE/EDEE**	RYGB (EDGE); Roux-en-Y hepaticojejunostomy and long-limb reconstructions (EDEE); anticipated repeat access	RYGB: EDGE technical 96%; Roux-en-Y hepaticojejunostomy and other long-limb reconstructions: EDEE technical 87.3%, clinical 93.8%	EDGE AEs 17–20%, perforation 4%; EDEE AEs 20%, 9.1% LAMS-related	Reversible after fistula closure; allows duodenoscope use and repeat interventions; LAMS migration in single session.
**LA-ERCP**	RYGB, particularly with gallbladder in situ	RYGB: procedural success 93–98.1%	AEs 18–28%; PEP 3–7%, surgical site infection 3–9%, perforation 2–4%.	Allows same-session cholecystectomy; requires general anesthesia, operating room and surgeon–endoscopist coordination

## Data Availability

No new data were created or analyzed in this study. Data sharing is not applicable to this article.

## Abbreviations

The following abbreviations are used in this manuscript:

AEs	Adverse Events
B-II	Billroth II gastrectomy
BE-ERCP	Balloon-Enteroscopy-assisted ERCP
BI	Billroth I gastrectomy
DBE	Double-Balloon Enteroscopy
EA-ERCP	Enteroscope-Assisted ERCP
EDEE	Endoscopic ultrasound-directed transenteric ERCP
EDGE	Endoscopic ultrasound-directed transgastric ERCP
ERCP	Endoscopic Retrograde Cholangiopancreatography
ESGE	European Society of Gastrointestinal Endoscopy
EUS	Endoscopic Ultrasound
EUS-AS	EUS-guided Antegrade Stenting
EUS-BD	EUS-guided Biliary Drainage
EUS-HGS	EUS-guided Hepaticogastrostomy
EUS-RV	EUS-guided Rendezvous
LA-ERCP	Laparoscopic-Assisted ERCP
LAMS	Lumen-Apposing Metal Stent
OAGB	One-Anastomosis Gastric Bypass (mini gastric bypass)
PTBD	Percutaneous Transhepatic Biliary Drainage
RV	Rendezvous
RYGB	Roux-en-Y Gastric Bypass
SAA	Surgically Altered Anatomy
SBE	Single-Balloon Enteroscopy
SEMS	Self-Expandable Metal Stent
